# Recent population genomic insights into the genetic basis of arsenic tolerance in humans: the difficulties of identifying positively selected loci in strongly bottlenecked populations

**DOI:** 10.1038/s41437-019-0285-0

**Published:** 2019-11-27

**Authors:** Mario Apata, Susanne P. Pfeifer

**Affiliations:** Center for Evolution & Medicine, School of Life Sciences, Arizona State University, Tempe, AZ 85821 USA

**Keywords:** Population genetics, Evolutionary genetics

## Abstract

Recent advances in genomics have enabled researchers to shed light on the evolutionary processes driving human adaptation, by revealing the genetic architectures underlying traits ranging from lactase persistence, to skin pigmentation, to hypoxic response, to arsenic tolerance. Complicating the identification of targets of positive selection in modern human populations is their complex demographic history, characterized by population bottlenecks and expansions, population structure, migration, and admixture. In particular, founder effects and recent strong population size reductions, such as those experienced by the indigenous peoples of the Americas, have severe impacts on genetic variation that can lead to the accumulation of large allele frequency differences between populations due to genetic drift rather than natural selection. While distinguishing the effects of demographic history from selection remains challenging, neglecting neutral processes can lead to the incorrect identification of candidate loci. We here review the recent population genomic insights into the genetic basis of arsenic tolerance in Andean populations, and utilize this example to highlight both the difficulties pertaining to the identification of local adaptations in strongly bottlenecked populations, as well as the importance of controlling for demographic history in selection scans.

## Introduction

Arsenic, a naturally occurring element, is acutely toxic to humans. Although low levels of environmental exposure to arsenic are generally considered safe, epidemiological studies indicate that exposure to elevated levels represents an important global public health issue, impacting tens of millions of individuals annually, particularly in developing areas (Naujokas et al. 2013). As arsenic exposure in human populations is the result of both natural and anthropogenic causes, the temporal extent of exposure differs greatly between global populations. Capitalizing on these differences, recent studies have utilized population genomic approaches to begin to unravel the genetic underpinnings of arsenic tolerance in several populations. For example, particular Andean populations show evidence of exposure to arsenic-contaminated drinking water spanning the past 7000 years, and genetic changes have recently been correlated with an increased arsenic metabolization efficiency in these populations (Schlebusch et al. 2013; Apata et al. 2017). Yet, considerable challenges remain to meaningfully connect the measured phenotypic variance observed between individuals and populations, with causative genotypic variants, and ultimately fitness. We here review these recent results, contextualize them more broadly within the field of population genomics, and outline avenues for future research.

## A brief overview of global arsenic exposure and population-level phenotypic variation in humans

**Fig. 1:**
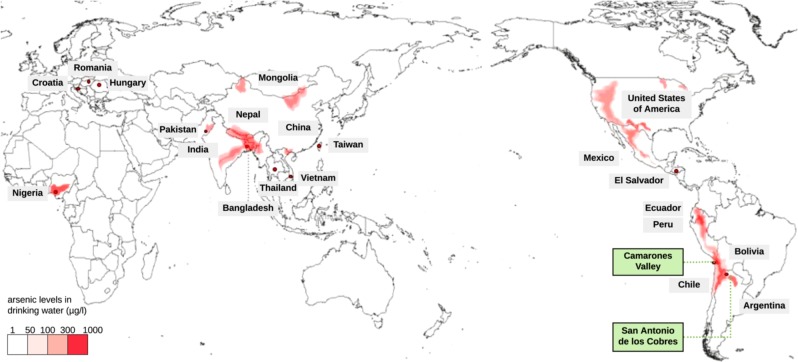
Map of endemic areas with high arsenic levels in natural water sources around the globe (Arriaza et al. 2018), with regions indicated on the red-scale frequently having significant public health issues associated with exposure. The regions denoted in green boxes have evidence of increased tolerance, perhaps owing to long-term exposure, and thus the local populations are fruitful examples in which to study the evolutionary response to arsenic

**Fig. 2:**
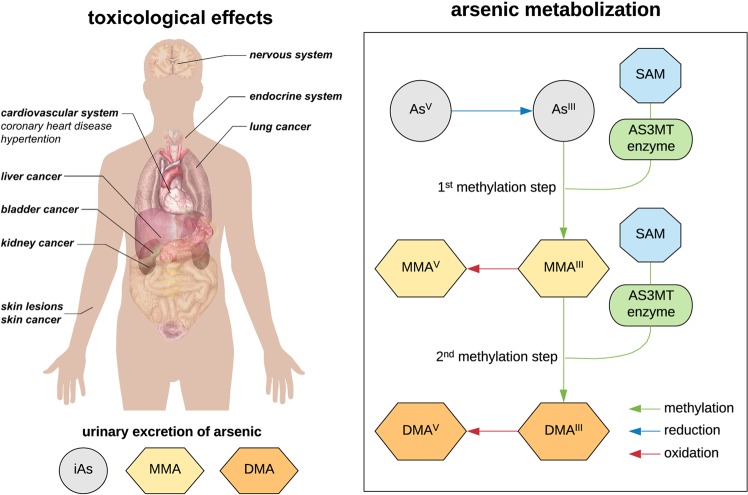
Toxicological effects on human health and the arsenic metabolization pathway. As described in the text, the relative proportion of monomethylarsonic acid (MMA) and dimethylarsinic acid (DMA) has arisen as a useful biomarker for predicting clinical outcomes. The figure is adapted from the ‘human body diagram’ image, released into the public domain by Mikael Häggström

Arsenic laden environments pose a serious public health issue, with current epidemiological crises in several countries along the Pacific Coast from South-East Asia to the Americas (Nordstrom 2002; Hughes et al. 2011). In these regions, over 90 million people are exposed to arsenic-contaminated water stemming from rivers, wells, and groundwater (Naujokas et al. 2013), with arsenic exposure ranging from 300 to 1000 µg/l (Fig. 1)—far surpassing 10 µg/l (i.e., the level considered safe by the WHO (2011)). This exposure owes to largely natural causes—with alluvial sediments, organic-rich or black shales, thermal springs, and volcanogenic sediments resulting in the dissolution of arsenic-bearing minerals (Nordstrom 2002; Fendorf et al. 2010; López et al. 2012). In addition, anthropogenic factors—for example, mining, mineral extraction, and agriculture (e.g., poultry and swine feed additives and pesticides), can contribute to an enrichment of arsenic in the groundwater (Nordstrom 2002). Chronic arsenic exposure has long been observed to result in genotoxic and carcinogenic effects, increasing morbidity owing to a high prevalence of several cancers in the skin, liver, and bladder (Argos et al. 2010) (Fig. 2). Furthermore, this element may pass through the placenta, increasing rates of spontaneous abortion, low birth weights, and several other conditions (Hopenhayn et al. 2003; Milton et al. 2017).

From an evolutionary perspective, it is firstly interesting to note that the general levels of arsenic tolerance, and consequently suffered toxicological effects, appear to vary between exposed populations across the globe, though the underlying evolutionary causes of these differences are only now beginning to be elucidated. As these toxicological and epidemiological effects have been well reviewed in the literature (see Minatel et al. 2018), we here particularly focus on the recent insights pertaining to the evolutionary response to long-term exposure, and discuss population genetic insights pertaining to the identified mutations observed to be associated with an increased arsenic tolerance.

Beginning with a broad view, most mammals use methyltransferases to metabolize inorganic arsenic into dimethylarsinic acid (DMA) via the highly toxic intermediate metabolite monomethylarsonic acid (MMA) (Fig. 2), though the ability to do so differs strongly across species (Palmgren et al. 2017). Within humans specifically, it is helpful to distinguish between populations experiencing short- vs. long-term exposure. In terms of the former, populations in Bangladesh are thought to have been exposed for only a few decades (Argos et al. 2010), and display characteristic symptoms including skin lesions as well as skin cancer (Pierce et al. 2011). In the Americas, short-term exposure, and the related effects, have been described in populations across the US, Mexico, and Latin America (McClintock et al. 2012). One of the most well-studied examples comes from northern Chile, in the city of Antofagasta. This population was shown to suffer severe effects upon the change of their primary drinking water source to the toxic Toconce River (arsenic level: 860 µg/l). The rapid increase in several arsenic-related diseases in this population, even 40 years after high exposures ceased, continues to be the subject of study (Ferreccio and Sancha 2006; Steinmaus et al. 2014; Roh et al. 2018).

Unlike these recent exposure events, Andean populations in South America have experienced a long-term exposure to arsenic. The Andeans are composed of several different groups including the Aymara, Atacameño, Quechua, and Colla, who have been living along the highlands and the Atacama Desert region (i.e., southern Peru, Bolivia, northern Chile, and Argentina) for thousands of years. Bio-archeological evidence suggests that pre-Columbian populations were likely suffering arsenic poisoning in the Atacama Desert at least 7000 years ago (Arriaza et al. 2010, 2018). Interestingly, modern populations from these regions have demonstrated reduced sensitivity to arsenic (Hopenhayn-Rich et al. 1996; McClintock et al. 2012), with certain diagnostic signs of chronic arsenic exposure (hyperkeratosis, hyperpigmentation, and skin cancer) being only rarely observed in the Aymara and Atacamaño populations (Sancha et al. 1992; Smith et al. 2000; De Loma et al. 2019). Indeed, recent studies indicate that modern Andean populations from the Camarones Valley of Chile, an area where arsenic levels in the water reach in excess of 200 µg/l, are able to metabolize arsenic efficiently and more rapidly expel it from their bodies via urination (Schlebusch et al. 2013; Apata et al. 2017).

Before addressing the genotypic underpinnings of this trait, it is first worth clarifying the nature of the phenotype. The primary detoxification pathway in humans occurs in the liver, during which MMA and DMA metabolites are produced (Fig. 2; Lin et al. 2002; Dheeman et al. 2014), and subsequently varying proportions of MMA, DMA, and inorganic arsenic are excreted (Agusa et al. 2011). The relative proportions differ between individuals and populations, and, as this variation is related to arsenic toxicity, this represents a sort of detoxification biomarker. Indeed, higher percentages of MMA are associated with various adverse health effects (Chen et al. 2003; Ahsan et al. 2006; Chung et al. 2009; Pierce et al. 2011, 2012), while higher levels of DMA are correlated with reduced toxicity. As expected, short-term exposed populations have higher MMA production, while cohorts from populations with long-term exposure have shown higher levels of DMA, including indigenous populations from Argentina and the highlands of Bolivia and Chile (Engström et al. 2011; Muñoz et al. 2018; De Loma et al. 2019).

## Connecting phenotype to genotype: searching for the genetic basis of efficient arsenic metabolization

Given the above phenotype, different genes encoding reductases and methyltransferases have been hypothesized to be the source of the inter-individual and inter-population variation in arsenic susceptibility (Fujihara et al. 2009, 2010; Agusa et al. 2011; Antonelli et al. 2014). For example, mutations in the genes *AS3MT* (arsenic [+3 oxidation state] methyltransferase), *GSTP1* (glutathione s-transferase pi 1), and *PNP* (purine nucleoside phosphorylase) have been found to alter metabolite profiles (Antonelli et al. 2014). Thereby, *AS3MT* has received particular attention owing to its key role in the catalysis of arsenic methylation (Sumi and Himeno 2012)—with a high methylation capacity being associated with higher levels of DMA and lower levels of MMA in urine (Gardener et al. 2011) (Fig. 2), and several mutations in this gene have been associated with related diseases (Antonelli et al. 2014).

**Fig. 3:**
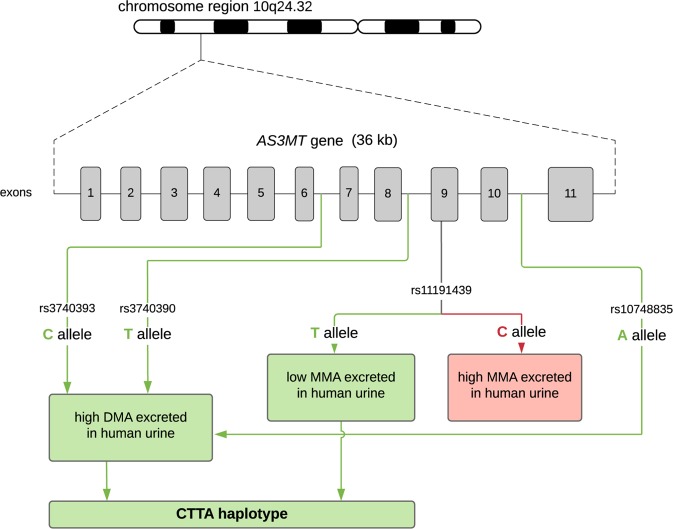
The identified haplotype in the *AS3MT* gene, found to be associated with increased arsenic tolerance. The C-T-T-A haplotype is a specific case of the C-T-A haplotype identified by Schlebusch et al. (2013) which, in addition to the three SNPs rs3740393, rs3740390, and rs10748835, also includes SNP rs11191439. Note that humans not carrying the identified haplotype have a higher toxicity risk

**Fig. 4:**
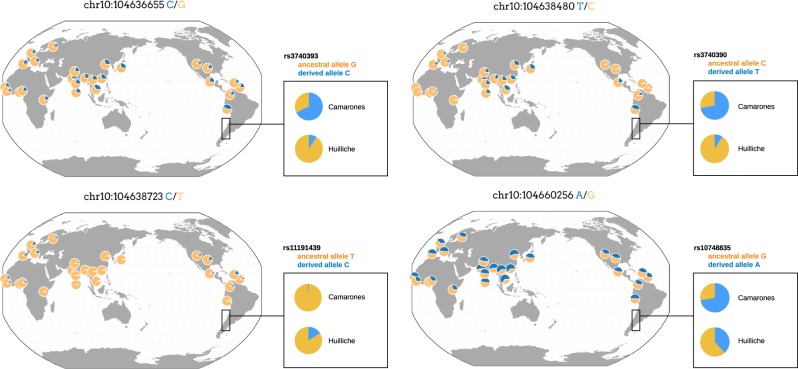
Population-specific allele frequencies for the four SNPs in the C-T-T-A haplotype in the *AS3MT* gene, found to be associated with increased arsenic tolerance. Allele frequencies for the Chilean populations (i.e., Camarones from the Camarones Valley and Huilliche from southern Chile) were obtained from Apata et al. 2017; allele frequencies for all other populations were obtained from the 1000 Genomes dataset (1000 Genomes Project Consortium 2015) using the Geography of Genetic Variants Browser (Marcus and Novembre 2017). Sites represent a comparison of regions with high and low levels of arsenic in the water, with levels reaching in excess of 200 µg/l in the Camarones Valley but less than 10 μg/l in southern Chile

Two polymorphic sites in particular have been strongly associated with efficient metabolization in modern populations exposed to arsenic (Agusa et al. 2011). The first is rs11191439 (Met287Thr), in which the ancestral T allele is associated with lower levels of the MMA metabolite. Previous studies in miners exposed to arsenic in Antofagasta have shown that carriers of the recessive C allele had higher percentages of MMA (Fig. 3), and thus a high toxicity risk (Hernández et al. 2008a, 2008b), with similar results from exposed populations studied in central Europe (Lindberg et al. 2007) and Mexico (Gomez-Rubio et al. 2012). The C allele shows a worldwide frequency around 10–14% (Fujihara et al. 2008, 2010), though is found at considerably lower frequency in populations experiencing long-term arsenic exposure (such as the 1% frequency observed in the Camarones) (Fig. 4). The second strongly associated variant is an intronic SNP, rs3740393 (C/G), which is at moderate frequency (35–40%) in short-term exposed populations (Fujihara et al. 2009, 2010), and higher frequency in long-term exposed populations (e.g., 70% in the Camarones; Fig. 4; Engström et al. 2011; Apata et al. 2017). CC and CG genotypes have been shown to be associated with an increase in the production of the DMA metabolite, and to be in strong linkage disequilibrium (LD) with two other intronic SNPs, rs3740390 (C/T), and rs10748835 (A/G). A joint haplotype of these three, known as C-T-A, is associated with higher percentages of DMA (Schlebusch et al. 2013), and is found at elevated frequency in the Camarones population (Apata et al. 2017).

Finally, the genomic region 10q24.32 has been generally associated with observed phenotypic variation in arsenic metabolization, with genome-wide association studies in Bangladesh reporting mutations associated with DMA/MMA variation (Pierce et al. 2012), consistent with associated studies in Andean populations associating these regions with higher DMA percentages (Engström et al. 2013; Schlebusch et al. 2015). In addition, this region has been highlighted as containing a potential signature of selective sweeps (Eichstaedt et al. 2015). However, while these mutations in *AS3MT* are interesting candidates, one must be careful when equating correlation with causation, and the population genetics of this trait and these genotypes are in need of further investigation, as discussed below.

## Putative signals of adaptation to arsenic: a case study in the Camarones Valley

Having colonized the Atacama Desert, one of the driest places in the world, around 9000 years ago, the first settlers faced environmental adversities ranging from high UV radiation to hypoxia to high arsenic concentrations in essentially all available drinking water. Yet, peoples from the pre-Columbian period to the present day have survived on this arsenic-contaminated water. The most exposed Andean population located in the Camarones Valley (arsenic level: 1000 µg/l) thus represents a unique natural laboratory to study the genetic underpinnings of any potential adaptation associated with arsenic-rich environments.

First considering bio-anthropological evidence, several studies have revealed severe arsenic concentrations in the inner organs, as well as visible skin lesions, in the remains of individuals from the ancient Chinchorro population—an Incan colony that was settled in the Camarones Valley (Arriaza et al. 2018). Arriaza (2005) posited that arsenic poisoning additionally increased rates of spontaneous abortion among the Chinchorro people, potentially initiating their characteristic artificial mummification practice, as an emotional and cultural response for coping with this loss. This hypothesis is partly based on the oldest ancient Chinchorro mummies (archeological sites of Cam-14 and Cam-17), corresponding predominantly to newborns and children (Arriaza 2005). Moreover, arsenic measures taken from hair and bones have shown a tendency of decreasing average arsenic levels over time, starting from Chinchorro hunter-gatherer-fishers in the Archaic Period (7000–3000 BP) to later agro-pastoral pre-Hispanic populations (3000–500 BP), to the current populations living in Quebrada Camarones (Yáñez et al. 2005; Arriaza et al. 2010; Byrne et al. 2010; Bartkus et al. 2011; Swift et al. 2015). This has been interpreted as a potential evidence of an adaptive, temporally increasing efficacy of metabolic detoxification. However, it should be noted that further genetic study is required to, for example, directly link the Chinchorro population as the ancestors of the modern Camarones population.

**Fig. 5:**
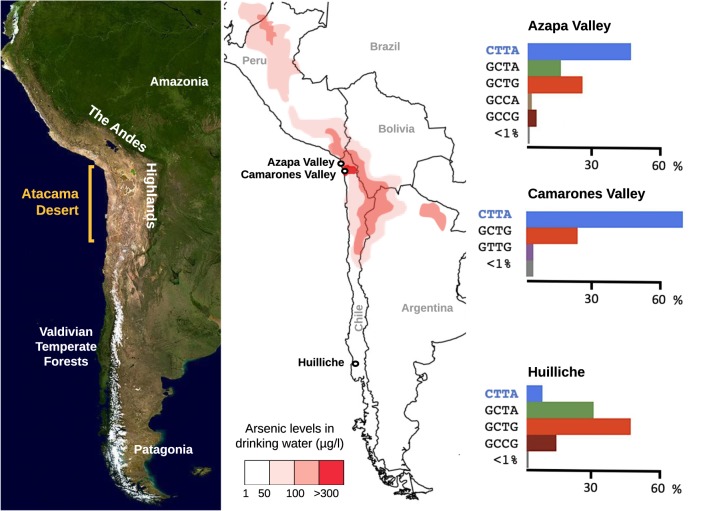
Observed haplotype frequencies in the *AS3MT* gene between Chilean populations with differing levels of arsenic exposure. The haplotype identified via association studies to be associated with increased arsenic tolerance, C-T-T-A, is shown in blue letters in the vertical axes of the graphs

Recently, a haplotype in the *AS3MT* gene (C-T-T-A—a specific case of the C-T-A haplotype previously identified by Schlebusch et al. (2013) which, in addition to the three SNPs rs3740393, rs3740390, and rs10748835, also includes rs11191439) has been identified in the Camarones population segregating at higher frequency (68%) than in other Amerindian populations with less arsenic exposure (the haplotype is found at 48% frequency in the Azapa Valley (arsenic levels: 10–20 µg/l), and 8% frequency in the Huilliche population (arsenic levels: <10 µg/l)) (Fig. 5). This observation, combined with the reduced frequency of the C risk allele in Met287Thr observed in long-term vs. short-term exposed populations (Camarones (1%) and Azapa (5%), vs. Hulliche (16%) and Antofagasta (14%)), has led to the suggestion that natural selection may be driving these observed frequency differences (Figs. 3–5).

However, while there indeed exist interesting phenotypic differences between long- and short-term exposed populations as described above, and though association studies have produced some promising candidate regions and mutations, considerable work remains in order to establish causal variants and to understand the evolutionary history of these populations. Without a proper population genetic framework accounting for the neutral processes shaping allele frequencies, including population size change, structure, and migration, inappropriate ‘adaptive story-telling’ remains a danger (Pavlidis et al. 2012; Jensen et al. 2019). In that regard, these small, admixed, highly bottlenecked indigenous populations present multiple challenges for distinguishing demographic and selective effects.

## The importance of distinguishing between population genetic signals of demography and selection

Strong population bottlenecks, of the sort believed to have been experienced multiple times throughout the evolutionary history of these populations (Lindo et al. 2018), are notoriously difficult to distinguish from genetic hitchhiking effects (Barton 1998; Thornton and Jensen 2007; and see the review of Bank et al. 2014). With regards to the particular methodologies thus far utilized in examining positive selection around the *AS3MT* locus in these Andean populations, Eichstaedt et al. (2015) relied on *F*_*ST*_ and PBS statistics (Yi et al. 2010), neglecting the demographic history and implementing a simple outlier approach. While common, such outlier approaches are fraught with error, owing to the a priori assumption that a set fraction of loci residing in the tail of a given statistical distribution must be owing to positive selection effects (i.e., neglecting the fact that any model, including neutrality, will have tails in the associated distribution). In addition, tenuous, the authors reported similarly significant *F*_*ST*_ values around this locus in populations without previous or current history of arsenic exposure. Moreover, in an earlier study, Eichstaedt et al. (2014) found that this genomic region was not significant with either the iHS or XP-EHH (Voight et al. 2006; Sabeti et al. 2007) approaches—suggesting a lack of evidence supporting the role hitchhiking effects modulating these allele frequencies (i.e., sweep-like patterns in LD do not appear to be present around this locus in these populations).

Similarly investigating putative patterns of positive selection around this locus, Schlebusch et al. (2015), considering population structure but not the history of population size change, found only weak support using the iHS statistic, and, like Eichstaedt et al. (2015), stronger evidence using a branch-length statistic (in this case, the LSBL statistics (Shriver et al. 2004)). Regardless of this mixed evidence, the performance of sweep-detection statistics under nonequilibrium demographic models is known to be severely comprised, often characterized by low power and high false-positive rates under a wide range of demographic scenarios (e.g., Jensen et al. 2005; Teshima et al. 2006; Excoffier et al. 2009; Bierne et al. 2011; Harris et al. 2018; and see the review of Crisci et al. 2012). Evaluating the performance of many of these statistics specifically, Crisci et al. 2013 found that both frequency spectrum and LD based approaches have false-positive rates often in considerable excess of true-positive rates, under strongly bottlenecked population histories such as these.

## Conclusions and future directions

Though results obtained without accounting for neutral processes are troubling in their implications regarding the ability to identify positively selected loci associated with arsenic exposure (or any other trait) in these populations, there is an upside. There exist increasingly sophisticated approaches for inferring demographic histories (e.g., Gutenkunst et al. 2009; Excoffier et al. 2013), which utilize high-quality, genome-wide data of the sort now available for these populations. While the resulting neutral demographic model may prove difficult to differentiate from selective effects as described above, the utilization of this model can at least greatly reduce mis-inference, and allow for the incorporation of the types of environmental measures that are available pertaining to arsenic exposure (see reviews of Haasl and Payseur 2016; Jensen et al. 2016). Fortunately, recent efforts have already begun to characterize the population history of this region, inferring population split times between low- and high-elevation populations in the Andes, as well as the severity of the population size reduction associated with European contact (Lindo et al. 2018).

As demographic inference continues to accumulate for these Andean populations, we propose a multistep process that can improve evolutionary inference in these populations in general, and with regards to the genotypic variants underlying phenotypic traits such as increased arsenic tolerance in particular. Having fit a demographic model, it is next of importance to assess the fit of that model to the genomic data. Namely, it is straightforward to simulate the parameters of the estimated model in order to ensure that it sufficiently replicates patterns of variation and LD observed in the natural population, using, for example, simulation software such as SLiM (Haller and Messer 2019). If the model is found to be a sufficient fit, one may then simulate selective sweeps of varying strengths and ages within the context of that demographic history. In such a way, by applying the planned statistical analyses (be it iHS, XP-EHH, *F*_*ST*_, or any other) to these simulated replicates, one may quantify the performance of that statistic (i.e., the true- and false-positive rates) under a population history actually relevant for the empirical application. With this information in hand, and if the results indeed suggest an ability to accurately detect selection for the population in question, genomic scan approaches may proceed. Relatedly, in the example of arsenic tolerance, given mixed evidence of selection on the candidate loci identified via association studies to date, as well as their unusual intermediate frequencies observed in both arsenic-exposed and nonexposed populations (Fig. 4; Eichstaedt et al. 2015), it appears worth also explicitly considering the potential of polygenic adaptation—a model which may not result in classic selective sweep signatures, and indeed may be simply characterized by subtle changes in allele frequency (Jain and Stephan 2017). However, differentiating these subtle frequency changes from those resulting from the underlying demographic history is expected to be even more challenging than for classic selective sweep models.

In closing, other systems in which genotype, phenotype, and fitness have been successfully connected may serve as useful models for future analyses pertaining to arsenic tolerance in humans. One of the best-known examples in mammals pertains to the evolution of cryptic coloration (see review of Harris et al. (2019)). As with the phenotypic observation of different levels of tolerance corresponding with the underlying levels of arsenic exposure in humans, in mouse populations the correspondence between coat coloration and soil color has long been noted (Dice 1947). In this instance, the correlation is likely owing to avian predation, in which cryptically colored individuals are more likely to avoid visual detection. Also, as in the arsenic example, large-scale association studies were conducted in order to identify genotypic variants that correlate with the phenotype in question (Linnen et al. 2009, 2013). As such, the cryptic coloration literature may serve as an informative roadmap outlining the avenues of future research. In that literature, genome-wide polymorphism data from light (derived-state) and dark (ancestral-state) mouse populations were next utilized to fit a demographic history including population size change, structure, and migration, and simulation was used to assess the fit of the model to the data and to characterize sweep-detection performance under the relevant demographic history (Pfeifer et al. 2018). Utilizing this model as the appropriate null distribution for characterizing the expected performance of summary statistics under neutrality compared to selection, well-supported candidate loci were identified. It is important to note, that certain mouse populations were found to have demographic histories characterized by bottlenecks of such severity that genomic scans for selection were simply not feasible, owing to the anticipated true- and false-positive rates (Poh et al. 2014). In populations for which genomic scans were applicable, statistically identified mutations were tested and functionally validated in *Mus*, thereby completing the link between genotype and phenotype using this mix of population genomics, association mapping, and functional assays (Barrett et al. 2019).

Thus, we propose that the way forward for the study of arsenic tolerance will involve more extensive demographic modeling, large-scale simulation studies to identify statistics that may be useful for identifying patterns associated with genetic hitchhiking/polygenic adaptation within the context of the inferred population history, and finally an examination of the correspondence between large-scale association studies and genomic scans in order to identify top candidate loci. With regards to the feasibility of functional studies to similarly validate candidates, it is noteworthy that tractable animal models indeed exist for the study of the *AS3MT* locus, with previous studies demonstrating its importance for arsenic methylation in rats (Lin et al. 2002) as well as in a mouse knockout (Drobna et al. 2009; Chen et al. 2011)—thereby providing potential study systems for the examination of existing and yet-to-be-identified candidate mutations.
